# Genetically engineered human induced pluripotent stem cells for the production of brain-targeting extracellular vesicles

**DOI:** 10.1186/s13287-024-03955-2

**Published:** 2024-10-08

**Authors:** Fan Tang, Tao Dong, Chengqian Zhou, Leon Deng, Hans B. Liu, Wenshen Wang, Guanshu Liu, Mingyao Ying, Pan P. Li

**Affiliations:** 1 Department of Psychiatry and Behavioral Sciences, Division of Neurobiology, Johns Hopkins University School of Medicine, 600 N. Wolfe St, Baltimore, MD 21287 USA; 2 Department of Radiology, Johns Hopkins University School of Medicine, Baltimore, Maryland USA; 3 Department of Neurology, Johns Hopkins University School of Medicine, Baltimore, Maryland USA; 4 Hugo W. Moser Research Institute at Kennedy Krieger, Baltimore, Maryland USA

## Abstract

**Background:**

Extracellular vesicles (EVs) are cell-secreted membrane vesicles that have become a promising, natural nanoparticle system for delivering either naturally carried or exogenously loaded therapeutic molecules. Among reported cell sources for EV manufacture, human induced pluripotent stem cells (hiPSCs) offer numerous advantages. However, hiPSC-EVs only have a moderate ability for brain delivery. Herein, we sought to develop a stable hiPSC line for producing EVs with substantially enhanced brain targeting by genetic engineering to overexpress rabies viral glycoprotein (RVG) peptide fused to the N terminus of lysosomal associated membrane protein 2B (RVG-Lamp2B) which has been shown capable of boosting the brain delivery of EVs *via* the nicotinic acetylcholine receptor.

**Methods:**

An RVG-Lamp2B-HA expression cassette was knocked into the AAVS1 safe harbor locus of a control hiPSC line using the CRISPR/Cas9-assisted homologous recombination. Western blot was used to detect the expression of RVG-Lamp2B-HA in RVG-edited hiPSCs as well as EVs derived from RVG-edited hiPSCs. Uptake of EVs by SH-SY5Y cells in the presence of various endocytic inhibitors was analyzed using flow cytometry. Biodistribution and brain delivery of intravenously injected control and RVG-modified EVs in wild-type mice were examined using ex vivo fluorescent imaging.

**Results:**

Here we report that an RVG-Lamp2B-HA expression cassette was knocked into the AAVS1 safe harbor locus of a control hiPSC line using the CRISPR/Cas9-assisted homologous recombination. The RVG-edited iPSCs have normal karyotype, express pluripotency markers, and have differentiation potential. Expression of RVG-Lamp2B-HA was detected in total cell extracts as well as EVs derived from RVG-edited (vs. control) hiPSCs. The RVG-modified EVs enter neuronal cells *via* distinct endocytic pathways, compared with control EVs. The biodistribution study confirmed that EVs derived from RVG-edited hiPSCs possess higher brain delivery efficiency.

**Conclusion:**

Taken together, we have established stable, genetically engineered hiPSCs for producing EVs with RVG expression, offering the improved ability for brain-targeted drug delivery.

**Supplementary Information:**

The online version contains supplementary material available at 10.1186/s13287-024-03955-2.

## Introduction

Extracellular vesicles (EVs) are cell-secreted membrane-surrounded vesicles with blood brain barrier (BBB) penetration potential, capable of delivering exogenous therapeutic molecules [1]. Human induced pluripotent stem cells (iPSCs) have been considered as one of the best sources for EV manufacture, based on (1) more abundant EV secretion than other cells, e.g., mesenchymal stem cells (MSCs) [2], (2) availability of cGMP-compatible clinical-grade manufacture platform for iPSC production [3, 4] and EV manufacture [5], (3) feasibility to perform genome editing to establish engineered iPSCs that produce modified EV for more efficient tissue-specific targeting [6, 7], and (4) option to generate personalized iPSC-derived EV from individual patients [8]. However, hiPSC-derived EVs only have a moderate ability for brain delivery.

The rabies viral glycoprotein (RVG) peptide was previously fused to the N terminus of lysosomal associated membrane protein 2B (Lamp2B) [9, 10], resulting in RVG peptide being displayed on the surface of EVs, leading to EV uptake by neuronal cells *via* the nicotinic acetylcholine receptor (nAChR) [11, 12]. Well-documented literature indicates that intravenous injection of RVG-modified EV delivered cargo (such as siRNA) specifically to the mouse brain [11–14]. More recently, RVG peptide was presented to the surface of EVs, *via* a CP05 peptide that binds to the second extracellular loop of CD63 protein with high affinity, for enhanced brain targeting of EVs [15]. However, an engineered cell line that stably expresses RVG-Lamp2B-HA and reliably produces RVG-modified EVs is still lacking.

Here, using CRISPR/Cas9 genome editing approach, we generated an hiPSC line that stably expresses RVG-Lamp2B from the safe harbor AAVS1 locus, and the EVs isolated from the RVG-edited hiPSCs exhibit improved brain targeting efficiency, compared with EVs isolated from the isogenic unedited control hiPSCs. Thus, we have established a stable, genetically engineered hiPSC line for producing RVG-modified EVs, which may be used for the brain-targeted delivery of therapeutic agents for the treatment of neurological disorders.

## Materials and methods

### Materials

Puromycin was purchased from Thermo Fisher. Cytochalasin D, wortmannin, and dynasore were purchased from Cayman Chemicals. Nystatin was purchased from Sigma-Aldrich. 3-(4,5-dimethylthiazol-2-yl)-2,5-diphenyltetrazolium bromide (MTT) and lactate dehydrogenase (LDH) assay kit were purchased from Invitrogen. Hoechst 33,342 was purchased from Cell Signaling Technology. Coomassie brilliant blue R-250 solution was purchased from Bio-Rad. Primary antibodies for OCT-4 A, SOX2, Nanog, SSEA4, TRA-1-60, TRA-1-81, NACM1, FOXA2, HA tag, Alix, CD63, CD81, CD9, Flotillin-1, Syntenin-1/MDA9, and β-actin were purchased from the Cell Signaling Technology. Primary antibodies for Sox17, Nestin, and PAX6 were purchased from Biolegend. Primary antibodies for nAChR were purchased from Abcam. Fluorophore-conjugated secondary antibodies, including AF-568 anti-rabbit IgG, AF-488 anti-rabbit IgG, and AF-488 anti-mouse IgG, were purchased from Thermo Fisher Scientific. HRP-linked secondary antibodies, including anti-rabbit IgG and anti-mouse IgG, are purchased from the Cell Signaling Technology.

### DNA plasmids

pAAVS1-P-CAG-DEST donor plasmid, containing 5’ and 3’ AAVS1 homologous arms (HAs) for recombination into the AAVS1 site was obtained from Addgene (ID: 80490[16]). RVG-Lamp2B-HA [9] sequence (1407 bp) was gene synthesized by Genscript and then cloned into pAAVS1-P-CAG-DEST plasmid at KpnI and BstXI sites in order to obtain pAAVS1-RVG-Lamp2B-HA donor plasmid. pX330-U6-Chimeric_BB-CBh-hSpCas9 plasmid was obtained from Addgene (ID: 42230[17]). pX330-AAVS1 T2 plasmid with gRNA sequence GGGGCCACTAGGGACAGGAT for human AAVS1 locus was obtained from Addgene (ID: 72833[18]).

### Cell culture

A control hiPSC line, CS25i-18n2, was obtained from Cedars Sinai Induced Pluripotent Stem Cell (iPSC) Core, and grown in StemMACS PSC-Brew XF medium (Miltenyi Biotec), on Geltrex substrate (Thermo Fisher) at 37 °C with 5% CO_2_. hiPSCs were passaged using gentle cell dissociation reagent (Stemcell Technologies) at 1:6 to 1:10 ratio when cells reached 80% confluence. The neuroblastoma SH-SY5Y cells (ATCC) were cultured in a 1:1 mixture of ATCC-EMEM and Gibco™ F-12 medium supplemented with 10% fetal bovine serum (FBS) and 100 U/ml penicillin-streptomycin, according to the ATCC protocol.

### CRISPR/Cas9-assisted homologous recombination

2 × 10^6^ human iPSCs were electroporated using the Celetrix electroporator, buffer and 120 µl pressured tubes (Celetrix) with 10 µg total of plasmids (4 µg of pAAVS1-RVG-Lamp2B-HA, 4 µg of pX330-AAVS1 T2 or pX330-U6-Chimeric_BB-CBh-hSpCas9, and 2 µg of pEF-BCL-XL [19]) at 620 V for a single pulse of 30 ms. After electroporation, the cells were immediately transferred to warm StemMACS PSC-Brew XF medium, and 1X RevitaCell supplement (Thermo Fisher) was added for 18 h to improve survival. Electroporated hiPSCs were passaged at a 1:6 ratio using gentle cell dissociation reagent whenever the culture reached confluence. Targeted hiPSCs were positively selected by 1 µg/mL puromycin treatment for 48 h from day 7 post electroporation. Surviving colonies were manually picked and expanded for culture and screened by junction PCR using F1/R1 and F2/R2 primers (Table S1). Genotyping PCR using F3/R3 primers [20] (Table S1) surrounding the gRNA region was used to determine if the single clone is homozygous or heterozygous for the integration into the *AAVS1* locus.

### Characterization of the RVG-edited iPSC line

Karyotyping and G-banding analysis was performed by WiCell cytogenetics, as previously described. Immunofluorescence staining using antibodies against pluripotent markers (Oct4 and Sox2, etc.) was conducted as previously described [21, 22]. STEMdiff™ Trilineage Differentiation Kit (Stemcell Technologies) was used to differentiate hiPSCs into three germ layers (ectoderm, mesoderm, and endoderm), following the manufacturer’s instructions. Germ layer markers (PAX6 and Nestin for ectoderm, Brachyury and NCAM for mesoderm, and Sox17 and FOXA2 for endoderm) were analyzed by immunofluorescence staining, as previously described [21, 22]. Cell images were taken by the Axio Observer 7 fluorescence microscope (Zeiss).

### EV extraction and characterization

EVs were extracted from the conditioned medium of unedited control or RVG-edited hiPSC lines of similar passage numbers (35–40) by sequential centrifugation, as previously described [15]. Conditioned medium was centrifuged at 500 ×g for 5 min to remove cell debris. The remaining supernatant was centrifuged at 10,000 ×g for 30 min, followed by 0.22 µM filtration using the Millex^®^ membrane. The medium was then ultracentrifuged at 100,000 ×g for 1 h to pellet EVs. The pellet was washed with cold PBS three times and then ultracentrifuged at 10,000 ×g for recovery. EV protein concentration was measured using Pierce™ BCA protein assay kit (Thermo Scientific). For characterization, EVs were measured in size by nanoparticle tracking analysis (NTA) and imaged by transmission electron microscopy (TEM). For NTA analysis, the NTA instrument (Particle Metrix) was calibrated by 100 nm polystyrene beads (Thermo Scientific) and set as following configurations: mode, scatter 488; sensitivity, 80; shutter, 70; frame rate, 69; track length, 15; Min brightness, 20; Min area, 10; cycle/positions, 2/11. PBS (that is filtered by a 0.22 µM membrane) was used as the NTA background control. In TEM analysis, 10 µl EV sample was loaded on immediately glow-discharged, copper carbon formvar grids. The grids were floated with EVs for 5 min and then washed, by floating the grids on the water drop, two times with each time for 30 s. The grids were then stained with 2% uranyl acetate, by floating the grids on the stain drop for 30 s. The grids were blotted off with Whatman™ papers and analyzed using the 80 kV 1230 TEM (Jeol).

### Western blotting analysis

Human iPSCs and EV samples were separately lysed with the RIPA lysis buffer supplemented with phenylmethanesulfonylfluoride, sodium orthovanadate, and protease inhibitor cocktail (Santa Cruz Biotechnology). Protein concentrations of iPSC and EV lysates were measured using the Pierce™ BCA protein assay kit. EV protein samples (30 µg) were loaded to Blot™ mini gel (MES) and gel electrophoresis was run at 200 V for 22 min. Gel was then transferred to the novex^®^ nitrocellular membrane with a wet transfer tank at 10 V for 60 min. The transferred membrane was blocked with 5% bovine serum albumin (BSA; Sigma-Aldrich) at RT for 1 h. The membrane was then incubated with different primary antibodies at 4 °C overnight. Next day, the membrane was washed with PBS three times for 5 min each time. Washed membrane was then probed with HRP-linked secondary antibodies at RT for 2 h. After washing with PBS three times again, the membrane was imaged and band intensities were quantified (Fig. 2D) using the Odyssey Fc Imager (Li-COR Biosciences).

### Neuronal uptake of EVs

EV protein cargo was labeled with green fluorescence using the ExoGlow™-protein EV labeling kit (System Biosciences). To analyze neuronal uptake of EVs, SH-SY5Y cells were seeded at 100,000 cells/well into a 24-well plate. The cells were incubated with labeled EVs (60 µg/µl) for 0 to 240 min at 37 °C. Incubated cells were washed by PBS and dissociated into single cells by trypsin EDTA (Gibco). Fluorescence of single cells was analyzed by the LSR II flow cytometer and the FlowJo software (Becton Dickinson). To visualize such uptake process, SH-SY5Y cells were grown at 30,000 cells/well in an 8-well chamber slide (Ibidi). Cell nuclei were stained using Hoechst 33342 (2 µg/ml) at 37 °C for 15 min. Labeled EVs (60 µg/µl) were added into cells, and time lapse recording of EV uptake by SH-SY5Y cells (that were maintained at 37 °C supplied by 5% CO_2_) was recorded for 240 min. For temperature-dependent analysis, SH-SY5Y cells were pre-incubated at 4–37 °C for 30 min and then incubated with labeled EVs for 1 h at the same temperature. Similarly, for endocytic pathway analysis, SH-SH5Y cells were pre-treated with cytochalasin D, wortmannin, nystatin, or dynasore for 30 min, and then cells were treated with labeled EVs, together with these inhibitors for 1 h. All cells were dissociated into singles cells by trypsin-EDTA and analyzed using the LSR II flow cytometer to record 5000 events/sample. Data were analyzed with the BD FlowJo™. To detect the colocalization of nAChR and RVG-Lamp2B, SH-SY5Y cells were seeded into an 8-well chambered slide (Ibidi), and the slide was incubated with labeled EVs at 37 °C for 1 h. Cells were then fixed with 4% paraformaldehyde at RT for 15 min. Fixed cells were permeabilized with 0.1% Triton™ three times. Permeabilized cells were blocked with 5% BSA at RT for 1 h, followed by incubation with rabbit anti-HA tag antibodies at 4 °C overnight. Next, cells were washed with PBS for three times and incubated with AF594 anti-rabbit IgG at RT for 2 h. Finally, the cells were washed with PBS again and visualized using LSM 880 confocal microscope (Zeiss).

### MTT and LDH assays

SH-SY5Y cells were seeded into a 96-well plate with 6 replicates at 10,000 cells/well. The cells were then treated with EVs at various doses for 24 h. MTT solution was incubated with cells at a final concentration of 0.5 mg/ml for 3 h. Formazan crystals formed in the MTT assay were dissolved with 100 µl DMSO (Sigma-Aldrich). The absorbance of DMSO solution was measured at 560 nm using the GloMax^®^ Discover microplate reader (Promega). For the LDH assay, the cell culture medium (50 µl) from EV-treated cells was incubated with an LDH reaction mixture at RT for 30 min. The incubation was protected from light and stopped by the stop solution (50 µl) according to product instructions. The LDH absorbance was measured at 490 nm using the GloMax^®^ Discover microplate reader.

### Brain-targeting and biodistribution of EVs

Wild-type (WT) mice were maintained in the C57BL6/J background and bred and maintained according to the Johns Hopkins University IACUC protocol, in accordance with National Institutes of Health guidelines. Control and RVG-modified EVs were labeled with the NIR dye using the ExoGlow™-Vivo EV labeling kit (System Biosciences). Labeled EVs were washed using PBS and recovered by ultracentrifugation at 10,000 ×g for 30 min. NIR labeled Control or RVG-modified EVs (250 µg) were intravenously injected into the tail vein of three-month-old WT female C57BL/6J mice. After 24 h, mice were deeply anesthetized using isoflurane, followed by transcardiac perfusion using PBS to remove EVs in blood circulation. No adverse events were observed, and each animal used had a humane endpoint. The mouse brain, liver, kidney, spleen, lung, and heart were then collected; the NIR fluorescence within each organ was imaged and analyzed using the Pearl^®^ imager (LI-COR Biosciences).

### Safety evaluation

Ten sex-matched WT C57BL/6J mice at the age of three months were randomly assigned to either the PBS control or EV treatment group. They were then intravenously administrated with PBS or 250 µg RVG-modified EVs every two days for seven times in total. After two weeks, these mice were sacrificed. Their whole blood, serum, and organs were collected. Blood chemistry, blood cell count, cytokine levels, and H&E staining histology analyses were then conducted as previously described [23, 24].

### Anesthesia and euthanasia of mice

For tissue and organ collection, C57BL/6J mice were anesthetized using a precision vaporizer supplied with 2% isoflurane at a gas flow rate of 1 L/min. The mouse’s nose was gently held against an anesthetic nose cone. The mouse anesthesia status was verified using toe-pinch reflex testing before surgical procedures. In safety evaluation, the treated mice were anesthetized using an open-drop method for retroorbital blood sampling. Specifically, a bottle containing 1/500 volume of isoflurane was prepared in a ducted hood. The mice were then accommodated above a wire mesh in the bottle to avoid contact between mice and the isoflurane. Mouse status was closely monitored to prevent under- or overdose of anesthesia. After sample collection, mouse euthanasia was conducted through carbon dioxide inhalation. Mice were grouped and euthanized in their home cage at a gas-filling rate of 50% per minute. The mice, after being unconscious, were maintained in the CO_2_ chamber for 5 min. Finally, their death was confirmed by cardiac and respiratory arrest. The animal experiments comply with standard operating procedure guidelines for animal research at Johns Hopkins University (JHU). These experiments have obtained the approval from the JHU Animal Care and Use Committee.

### Statistical analysis

At least three biological replicates of each experiment were performed. Data were analyzed using the GraphPad Prism 10 software and presented as mean ± SEM. The minimum sample size (*n*) was estimated by the effect size, SEM, study power, and confidence level for each experiment. Animal experiments do not involve data exclusion, randomization of allocation, or confounder minimization strategies. The results were analyzed using students’ t test for comparison between two groups, or one-way analysis of variance (ANOVA) followed by Tukey post hoc test for comparison between three or more groups. Statistical significance was set at the *p* value < 0.05.

## Results

### Genetic engineering of hiPSCs for the generation of RVG-modified EVs

**Fig. 1:**
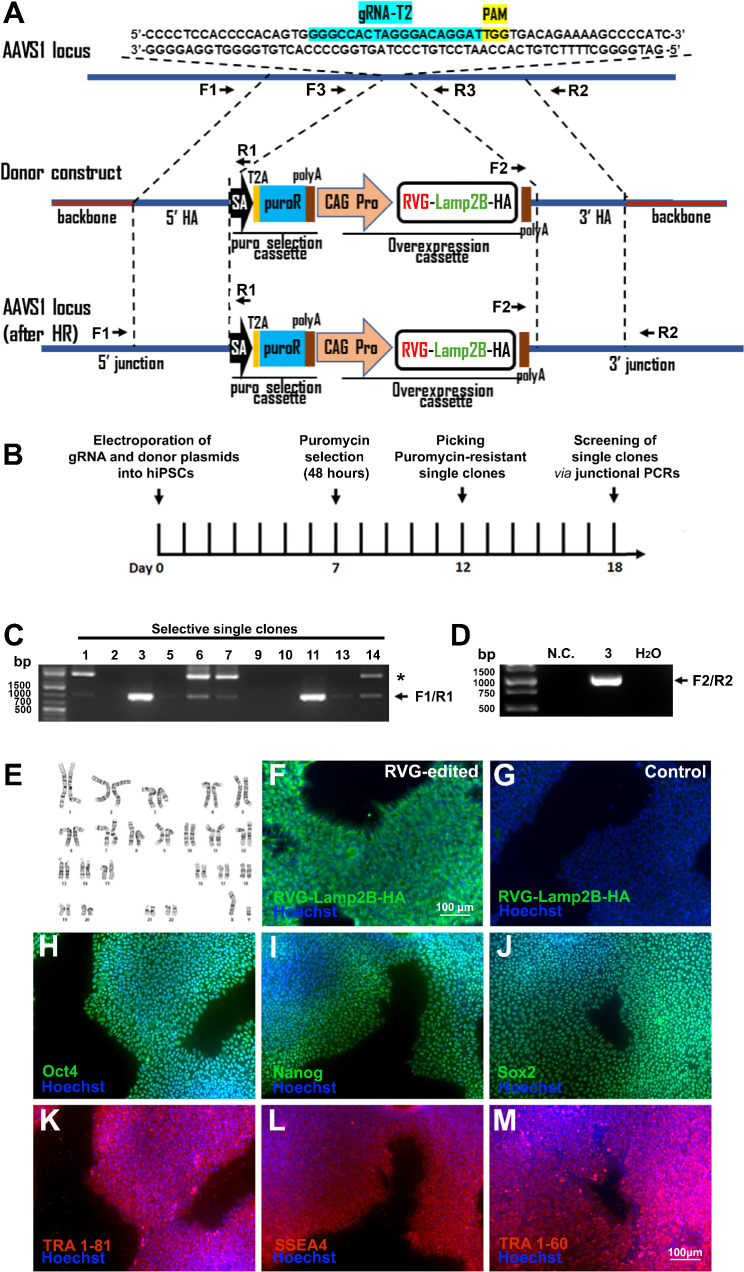
Generation of a genetically engineered hiPSC line that overexpresses RVG-Lamp2B-HA from the AAVS1 locus. (**A**) Schematic diagram (not drawn to scale) shows the AAVS1 locus before and after homologous recombination (HR). PAM: the protospacer-adjacent motif; SA: a splice acceptor; T2A: 2A self-cleaving peptides; puroR: a puromycin resistance gene; poly A: the poly-A tail for mRNA; HA: homologous arms; RVG-Lamp2B: RVG fused to the N-terminus of Lamp2B; CAG Pro: a CMV early enhancer/chicken β actin promoter. The donor construct includes an RVG-Lamp2B-HA overexpression cassette and a puro selection cassette. The expression of RVG-Lamp2B-HA is driven by a constitutive CAG promoter. (**B**) Timeline of genome editing, including puromycin selection and screening. (C-D) PCR-based screening of 5’ junctions to identify successfully targeted puromycin-resistant iPSC clones. ~800 bp band (primers F1/R1) indicates a successfully generated 5’ junction (**C**). ~1.1 kb band (primers F2/R2) indicates a successfully generated 3’ junction (**D**). Primer locations are as indicated in Fig. 1A; unincorporated donor DNA and junctional regions of unsuccessfully edited cells cannot be amplified by these primers. N.C.: negative control. Asterisk indicates a non-specific PCR band. The full-length gel image is presented in Fig. S8A. (**E–M**) Characterization of the RVG-edited iPSCs. (**E**) Karyotyping of RVG-edited hiPSCs shows no chromosomal abnormalities. (**F-G**) Anti-HA tag immunostaining detected overexpression of RVG-Lamp2B-HA in RVG-edited hiPSCs, but not in unedited control hiPSCs. (**H-M**) RVG-edited line expresses pluripotent markers Oct4 (**H**), Nanog (**I**), Sox2 (**J**), TRA1-81 (**K**), SSES4 (**L**), and TRA1-60 (**M**). Scale bar: 100 μm

To obtain an isogenic human iPSC line that overexpress RVG-Lamp2B-HA, a ~ 4 kb DNA fragment (Fig. 1A), including an RVG-Lamp2b-HA [9] expression cassette and a puromycin selection cassette, was successfully knocked into the *AAVS1* safe harbor locus of a control hiPSC line, CS25i-18n2, using the CRISPR/Cas9-assisted homologous recombination. The genome editing strategy, along with the timeline, is outlined in Fig. 1A and B. Two out of 13 puromycin selected clones were tested positive by junctional genotyping PCRs using primers F1 and R1 (Fig. 1C), and clone #3 was confirmed to be positive by junctional genotyping PCR using primers F2 and R2 (Fig. 1D). PCR amplification of the *AAVS1* locus with primers surrounding the gRNA (F3 and R3) indicated that clone #3 is heterozygous (Fig. S1A), and Sanger sequencing of the PCR product (F3 and R3) showed that indels were not introduced in the second allele of *AAVS1* in clone #3 (Fig. S1B-C). RVG-edited single clone #3 was chosen for subsequent experiments. The RVG-edited iPSCs have normal karyotype (Fig. 1E). Anti-HA immunostaining detected RVG-Lamp2B-HA expression in the isogenic RVG-modified hiPSCs (Fig. 1F), but not in the unedited control cells (Fig. 1G). The RVG-edited iPSCs express pluripotency markers, such as Oct4, Nanog, Sox2, TRA 1–81, SSEA4, and TRA1-60, as shown by immunostaining (Fig. 1H-M), and they exhibit potential to be differentiated into cells resembling three germ layers (Fig. S2). These results indicate that the genome editing procedure that was used to obtain the isogenic RVG-edited hiPSCs did not compromise the pluripotency or differentiation potential of the hiPSCs. To ascertain that the RVG-edited cells do not contain any insertion or deletion (indel) caused by the gRNA off-target effects, the top 5 potential off-target sites were predicted [22], and verified by PCR and Sanger sequencing (Fig. S3), the results of which indicate that the AAVS1 gRNA sequence used did not exhibit off-target effects. Thus, using genome editing, we have successfully established a stable RVG-edited hiPSC line that overexpresses RVG-Lamp2B-HA.

### Isolation and characterization of RVG-modified EVs

**Fig. 2:**
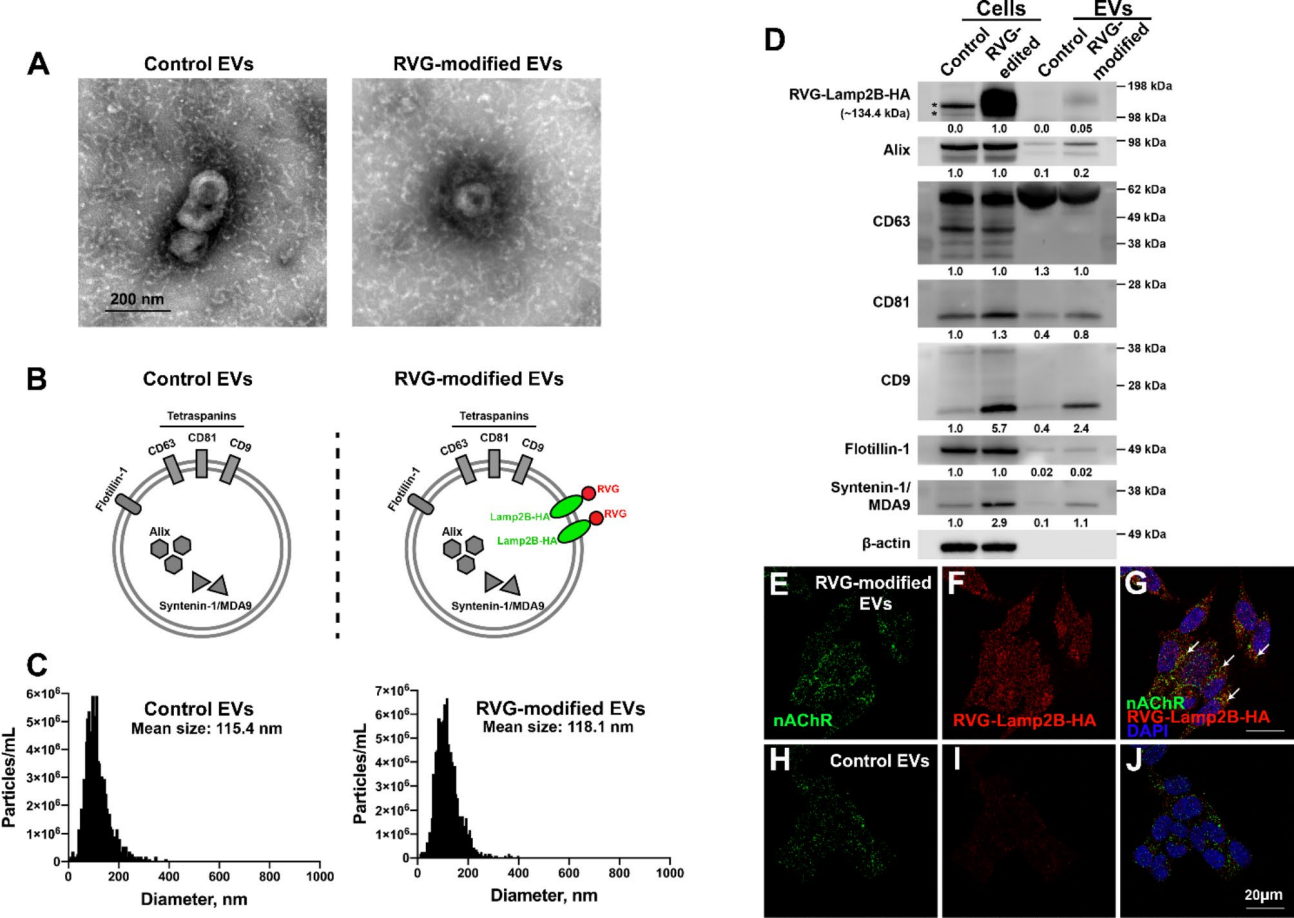
Characterization of control and RVG-modified EVs. (**A**) Transmission electron micrographs (TEM) and protein marker diagrams of EVs. TEM shows that control and RVG-modified EVs are in a cup-shaped morphology. (**B**) Unlike the control EVs, the RVG-modified EVs display RVG on the EV surface for nAChR binding. (**C**) Nanoparticle tracking analysis of Exo size. RVG modification increased the Exo size from 115.4 nm to 118.1 nm. (**D**) Western blotting analysis of protein markers in RVG-modified and control EVs together with their corresponding iPSC lines. RVG modification changed protein expression of RVG-modified EVs. Asterisks indicate non-specific bands from anti-HA western blot. Fold changes of exosomal marker proteins are shown. Full-length blots are presented in Fig. S8B. (**E-J**) Confocal colocalization of RVG-Lamp2B-HA (on RVG-modified EVs; detected using anti-HA antibodies) and nicotinic acetylcholine receptors (nAChR; detected using anti-nAChR antibodies) in SH-SY5Y cells. White arrows point to colocalization areas

To characterize the EVs derived from RVG-edited hiPSCs (RVG-modified EVs), in comparison with the EVs derived from the control unedited hiPSCs (control EVs), we extracted EVs from conditioned media of RVG-edited as well as the control unedited hiPSCs *via* sequential centrifugation as previously described [15]. Coomassie brilliant blue R-250 staining shows that CD63, as the major exosomal marker, has the highest expression in all EV protein extracts, indicating the high purity of our EV samples (Fig. S4). Transmission electron microscopy (TEM) reveals the saucer-shaped morphology for EVs derived from both unedited and RVG-edited hiPSCs, indicating that RVG modification did not impair morphological integrity of EVs (Fig. 2A). Schematic presentations of control and RVG-modified EVs are shown in Fig. 2B. Nanoparticle tracking analysis (NTA) reveals that RVG modification on the EVs increased the mean size by 2.7 nm, from 115.4 nm in unedited control EVs to 118.1 nm in RVG-modified EVs (Fig. 2C). Western blotting analysis shows that while the RVG-Lamp2B-HA is not expressed in unedited control hiPSCs or EVs, the amount of RVG-Lamp2B-HA expressed in RVG-modified EVs is approximately 5% of that expressed in the RVG-modified hiPSCs (Fig. 2D), presumably due to the fact that Lamp2B, as a lysosome-associated membrane protein, is primarily expressed and functions within cells rather than in the EVs secreted into the extracellular space. Exosomal protein markers, including alix, CD64, CD81, CD9, flotillin-1, and syntenin-1/MDA9, were expressed in both unedited control and RVG-modified cell and EV samples (Fig. 2D). Interestingly, all the exosomal markers tested, except for flotillin-1, are differentially expressed in the RVG-modified cell and EV samples, compared with the unedited control samples (Fig. 2D). Consistent with the expression of RVG-Lamp2B in RVG-modified EVs, immunostaining followed by confocal microscopy analysis shows that RVG-Lamp2B-HA on the RVG-modified EVs is colocalized with nicotinic acetylcholine receptor (nAChR) in SH-SY5Y cells (Fig. 2E and G), while this colocalization was not observed for unedited control EVs (Fig. 2H and J), indicating the nAChR binding property of RVG-modified EVs.

### Neuronal uptake of RVG-modified EVs via various endocytic pathways

**Fig. 3:**
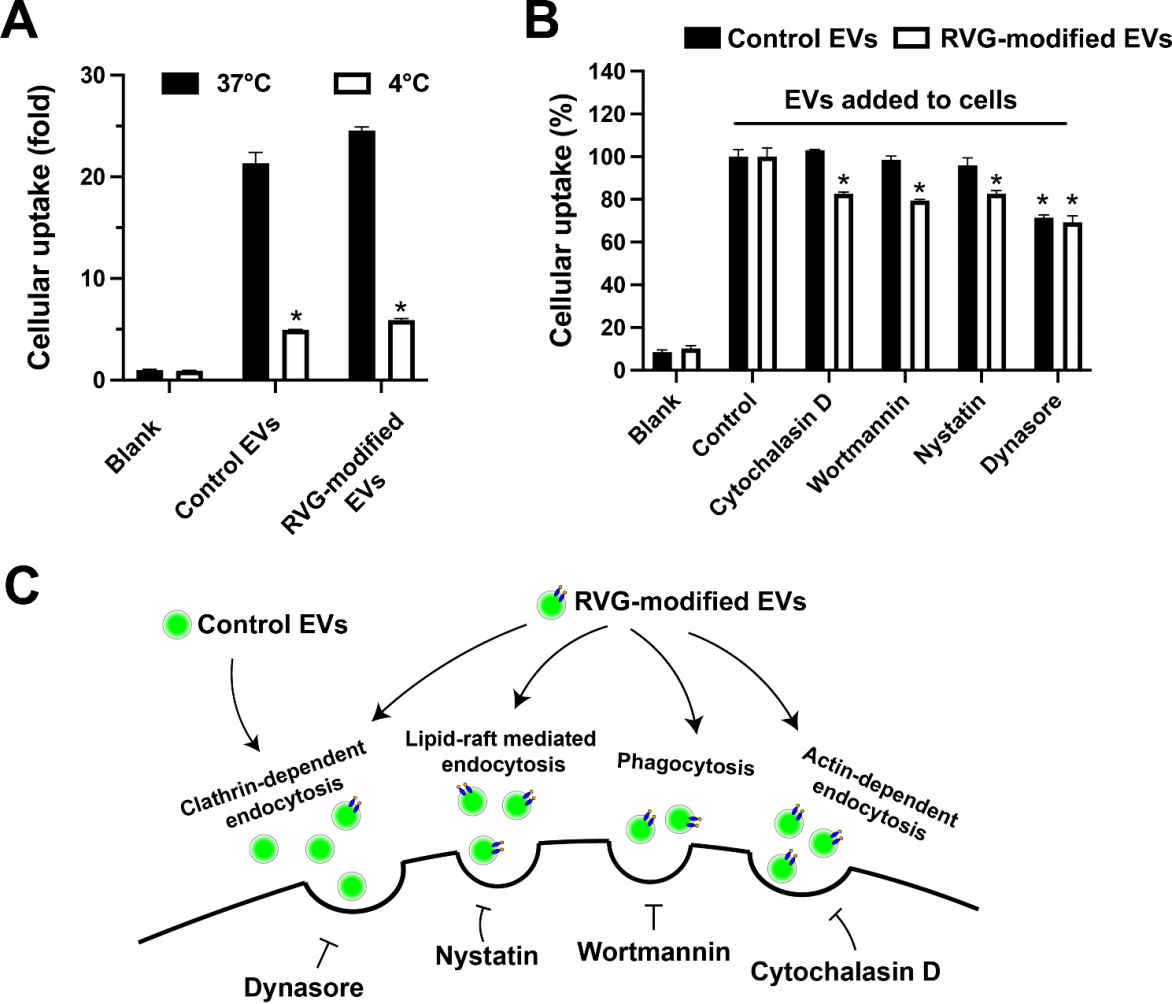
RVG-modified EVs enter neuronal SH-SY5Y cells *via* diverse endocytic pathways. (**A**) Cellular uptake of EVs at different temperatures after 1 h incubation as determined by flow cytometry. (**B**) Cellular uptake of EVs after 30 min treatment with endocytic inhibitors, including cytochalasin D for actin-dependent general endocytosis, wortmannin for phagocytosis, nystatin for lipid raft, and dynasore for clathrin-coated pit. Data are presented as mean ± SEM (*n* = 4 per condition; total *n* = 24 or 48) and analyzed using one-way ANOVA followed by Tukey post hoc test. *p < 0.05. (**C**) Schematic illustration for endocytic mechanisms of control and RVG-modified EVs

Next, we labeled EV protein cargo with green fluorescence and incubated the labeled control and RVG-modified EVs with SH-SH5Y neuroblastoma cells. Cellular fluorescence intensity was used as the outcome to examine the effect of RVG-modification on the neuronal uptake of EVs. Labeling the protein cargo, rather than the EV membrane, diminishes the influence of dye on membrane physicochemical properties, which may be critical for the interaction between EVs and plasma membrane during cellular uptake. Nano flow cytometry analysis suggests that RVG-modified EVs possess higher fluorescence intensity than the unedited control EVs, indicating that RVG-modified EVs have a higher labeling efficiency, compared with the control EVs, presumably due to upregulation of certain EV cargo proteins in RVG-modified EVs (Fig. S5A). The differential labeling efficiency between RVG-modified EVs and control EVs rendered it impossible to compare the neuronal uptake rate between control and RVG-modified EVs directly, but rather the uptake of either control or RVG-modified EVs under various conditions was normalized to that of itself under untreated condition, for the following analyses. The neuronal uptake of both control and RVG-modified EVs at 4 °C is decreased compared with that at 37 °C, suggesting that EVs enter cells in an energy-dependent manner (Fig. 3A). The differential expression of exosomal markers in RVG-modified EVs (Fig. 2D) potentially confers some novel biological properties compared with the control EVs, and we therefore hypothesize that RVG-modified EVs may enter the cells *via* differential endocytic pathways. Consistent with our hypothesis, endocytic inhibition analysis shows that cellular uptake of RVG-modified EVs can be inhibited by multiple endocytic inhibitors, including cytochalasin D for actin-dependent general endocytosis, wortmannin for phagocytosis, nystatin for lipid raft, and dynasore for clathrin-coated pit, while in contrast, the control EVs are mainly inhibited by dynasore (Fig. 3B). Therefore, our results suggest that RVG-modified EVs possess novel and diverse cell entering capacity compared with the control EVs (Fig. 3C), and this capacity potentially confers the RVG-modified EVs greater neurotropism in the brain.

### RVG-modified EVs exhibit enhanced brain-targeting properties in vivo

**Fig. 4:**
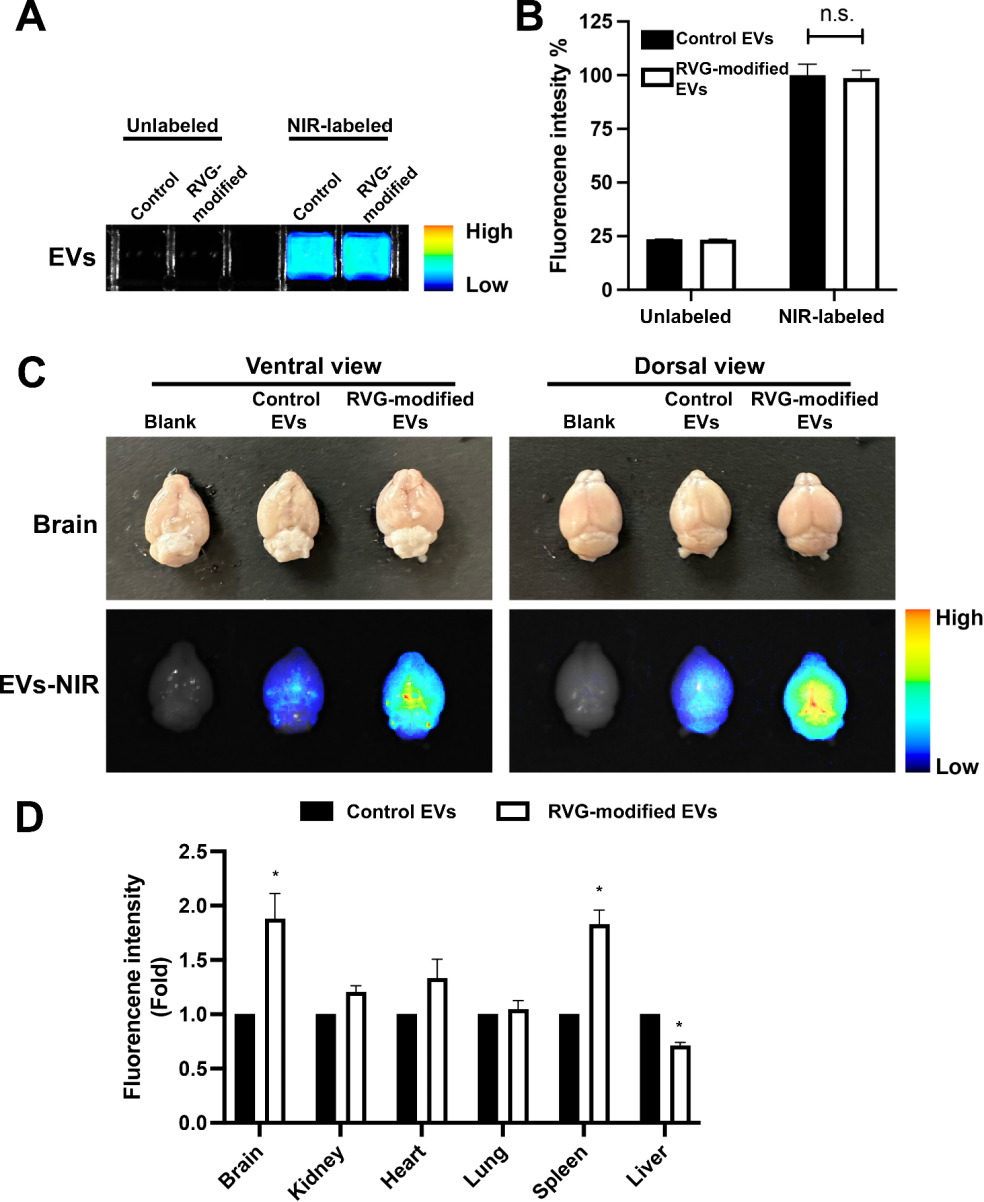
Enhanced brain-targeting capacity and biodistribution of RVG-modified EVs. (**A-B**) Control and RVG-modified EVs were labeled with NIR dye on their EV membrane with equivalent labeling efficiency. The fluorescence intensities of NIR dye labeled control EVs were normalized to 100%. (**C-D**) NIR dye labeled EVs were intravenously injected into C57BL/6J mice *via* the tail vein. After 24 h, mouse organs were imaged using the ex vivo fluorescence imaging system. RVG-modified EVs exhibit significantly higher NIR signals in the mouse brains compared with the control EVs (**C**). Biodistribution of control and RVG-modified EVs in different organs of the mice 24 h post intravenous injection (**D**). The NIR signals in the same organs from mice injected with control EVs were normalized to 1. Data were presented as mean ± SEM (*n* = 3 per condition; total *n* = 9) and analyzed using one-way ANOVA followed by Tukey post hoc test. *p < 0.05

To examine if RVG-modified EVs have improved brain-targeting properties compared with the control EVs, an equal amount of the control and RVG-modified EVs were labeled with a non-lipophilic NIR dye (Fig. 4A) and then intravenously injected into C57BL/6J mice *via* tail veins. Figure 4B shows equivalent dye labeling efficiency for both control and RVG-modified EV samples. At 24 h post systemic administration, mice were perfused with PBS to remove blood in circulation, various tissues including brain, kidney, lung, heart, spleen, and liver were collected, and fluorescence intensity within each organ was quantified as a readout for tissue-specific targeting. Ex vivo imaging reveals that fluorescence intensity in the brains of mice treated with the labeled RVG-modified EVs is over 1.5-fold higher than that from brains treated with the labeled control EVs (Fig. 4C), indicating that the RVG-modified EVs exhibit enhanced brain-targeting capacity. Ex vivo imaging analysis of peripheral tissues suggests that compared with the control EVs, the RVG-modified EVs accumulate less in liver, but more in brain and spleen (Fig. 4D and Fig. S6).

### Safety evaluation of RVG-modified EVs

**Fig. 5:**
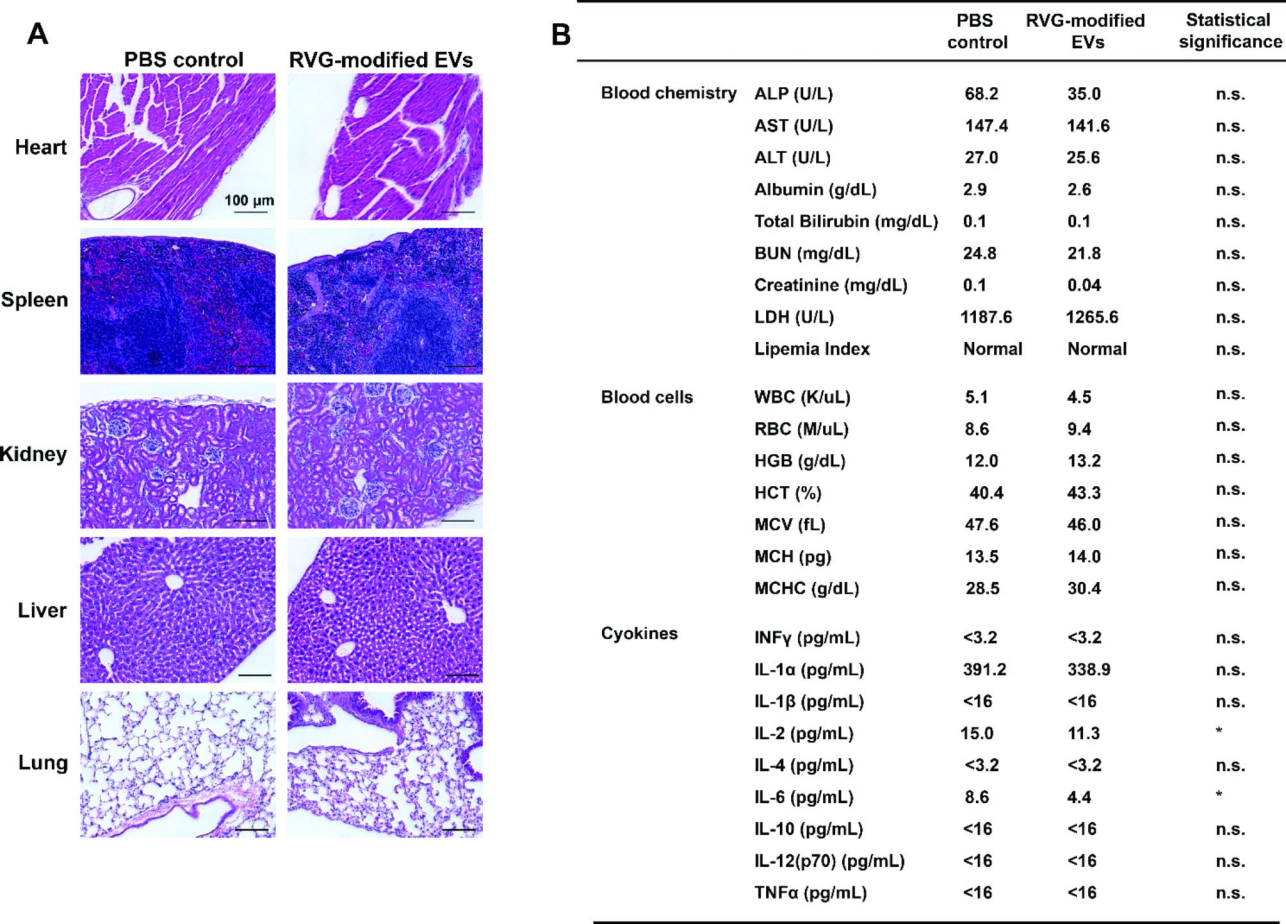
Biocompatibility of RVG-modified EVs in vivo. (**A**) H&E staining of the heart, spleen, kidney, liver, and lung from C57BL/6J mice intravenously injected with phosphate-buffered saline (PBS) or 250 µg RVG-modified EVs for 14 days (once every two days). (**B**) Blood chemistry and blood cell analysis suggest no difference between PBS and RVG-modified EVs groups. Although the ALP decreased (a less common observation), this decrease did not reach statistical significance. Serum cytokine analysis was conducted using antibody-based multiplex beads. Unlike other cytokines that fell beyond the detection range (i.e., < 3.2 or < 16), the IL-2 and IL-6 were decreased, suggesting the anti-inflammatory potential of the EVs. Data were presented as mean ± SEM (*n* = 5 per condition; total *n* = 10) and analyzed using one-way ANOVA followed by Tukey post hoc test. *p < 0.05

To systematically evaluate biocompatibility of RVG-modified EVs, we intravenously injected 250 µg RVG-modified EVs, or PBS control, into CS57BL/6J mice once every two days for seven times in total. Histology analysis suggested that RVG-modified EVs cause no tissue damage in heart, spleen, kidney, liver, and lung (Fig. 5A). Blood chemistry analysis showed that RVG-modified EVs do not alter serum components, including aspartate aminotransferase (AST), alanine aminotransferase (ALT), albumin, total bilirubin, blood urea nitrogen (BUN), creatinine, and lactate dehydrogenase (LDH) (Fig. 5B). Similarly, we found that RVG-modified EVs do not affect blood cell number or indexes (Fig. 5B). Since the potential reactogenicity to the RVG peptide presented on EV surface remains a potential concern, we measured cytokine levels in blood after systemic administration of RVG-modified EVs for two weeks. Our results showed that RVG-modified EVs significantly decrease IL-2 and IL-6 cytokine levels, slightly decrease the IL-1α level in CS57BL/6J mice. Other cytokine levels, i.e., GM-CSF, INFγ, IL-1 β, IL-4, IL-10, IL-12 (p70), and TNF α, were all below the level of detection in the blood samples collected from mice treated with PBS control or RVG-modified EVs (Fig. 5B), suggesting RVG-modified EVs did not upregulate these cytokines in vivo significantly. Overall, our results indicate that RVG-modified EVs are biocompatible in vivo, and have anti-inflammatory potential.

Consistent with previous reports regarding the neuroprotective effects of EVs, 3-(4,5-dimethylthiazol-2-yl)-2,5-diphenyltetrazolium bromide (MTT) assay suggests that labeled RVG-modified and control EVs increase cellular metabolism without cytotoxicity (Fig. S7A). Similarly, lactate dehydrogenase (LDH) release assay shows lower LDH release upon treatment of SH-SY5Y cells with either control or RVG-modified EVs, indicating that the cell membrane integrity is increased by EV treatment (Fig. S7B).

## Discussion

In this study, using CRISPR-assisted homologous recombination, we have generated a genetically engineered hiPSC line that constitutively expresses RVG-Lamp2B-HA. This engineered hiPSC line produces EVs that express RVG-Lamp2B and exhibit enhanced brain-targeting capacity when systemically administered into mice.

It is interesting that overexpression of RVG-Lamp2B regulated the expression of EV proteins or modified the protein “barcode” on the EV surface. This modification potentially confers RVG-modified EVs some novel biological properties other than nAChR binding. One potential explanation is that RVG modification on EVs changes expression of EV membrane proteins (Fig. 1D), important components that can determine tissue-targeting specificity of EVs. It is also likely that RVG modification might alter other membrane components, like phospholipids, by regulating intracellular pathways for protein lipidation. Indeed, we found that the RVG-modified EVs distinctively adopt diverse endocytic pathways during neuronal uptake. Besides, the unique barcode might account for reduced affinity of EVs to liver, a major metabolism organ for clearing therapeutic agents. Together with reduced liver retention and higher brain-targeting efficiency, the RVG-modified EVs can open up a new avenue for brain-targeted delivery, especially for drugs that may potentially cause liver damage. Generation of a control hiPSC line expressing Lamp2B-HA in the *AAVS1* locus in the future would help clarify the effect of RVG-Lamp2B-HA vs. Lamp2B-HA on the properties of hiPSC-derived EVs, such as differential expression of EV proteins and tissue targeting.

EVs in this study were isolated by sequential centrifugation [15]. The key exosomal marker, CD63, dominates protein components of the EVs isolated and indicates exosomes are a major subpopulation of isolated EVs. EV subpopulation mainly includes ectosomes and exosomes that are classified based on their site of biogenesis [25]. However, current isolation strategies, based on size, density, and affinity, insufficiently discriminate them. Heterogenicity of EV remains to be elucidated. Interference with specific biogenetic processes of EVs might offer us the knowledge of determinants for such heterogenicity.

Apart from exosomal proteins, routes of EV internalization used by recipient cells are important for EV biodistribution or organotropism. So far, major routes are reported to be endocytosis and direct membrane fusion [26]. Our results show that RVG-modified EV crosses the cell membrane *via* diverse endocytic pathways, while the control EV mainly enters *via* the clathrin-coated pit pathway. It is likely that the binding between RVG-modified EV and nAChR drives intracellular signaling to finalize EV entry pathways in neuronal cells, and that diversity in cellular uptake pathways could further enhance the neurotropism of RVG-modified EVs.

In conclusion, using CRISPR/Cas9 genome editing, we have successfully established an RVG-engineered hiPSC line for the production of RVG-modified EVs. When compared with the control unedited EVs, RVG-modified EVs show significantly higher brain tropism in mice. Thus, this study may open up a new avenue for the development of brain-targeting drug carriers, which may be applied to the treatment of various neurological disorders.

## Electronic supplementary material

Below is the link to the electronic supplementary material.

Supplementary Material 1

## Data Availability

The data supporting the results reported in this manuscript are archived in the Figshare repository. The DOI is 10.6084/m9.figshare.25889455.

## Declarations

### Protocol registration

No protocol was prepared and registered before this study.
