# Supplementary material for: Genetically engineered human induced pluripotent stem cells for the production of brain-targeting extracellular vesicles
Source: Stem Cell Res Ther. 2024 Oct 8;15:345. doi: 10.1186/s13287-024-03955-2 (PMC11462716; doi:10.1186/s13287-024-03955-2)
Supplement: Supplementary file 1 — Supplementary Material 1 [file 13287_2024_3955_MOESM1_ESM.docx]

**Title**

Genetically engineered human induced pluripotent stem cells for the production of brain-targeting extracellular vesicles.

Authors:

Fan Tang^1^, Tao Dong^1^, Chengqian Zhou^1^, Leon Deng^1^, Hans B. Liu^1^, Wenshen Wang^2,4^, Guanshu Liu^2,4^, Mingyao Ying^2,4^, Pan P. Li^1^*

Addresses:

^1^Department of Psychiatry and Behavioral Sciences, Division of Neurobiology, Johns Hopkins University School of Medicine, Baltimore, Maryland, USA

^2^Department of Radiology, Johns Hopkins University School of Medicine, Baltimore, Maryland, USA

^3^Department of Neurology, Johns Hopkins University School of Medicine, Baltimore, Maryland, USA

^4^Hugo W. Moser Research Institute at Kennedy Krieger, Baltimore, Maryland, USA

*Corresponding Author

Pan P. Li

Department of Psychiatry and Behavioral Sciences, Division of Neurobiology, Johns Hopkins University School of Medicine, 600 N. Wolfe St., Baltimore, MD 21287, USA

Telephone: +1 4105023760; Fax: +1 4106140013; Email: [ple5@jhmi.edu](mailto:ple5@jhmi.edu)

Relevant conflict of interest/financial disclosure: Nothing to report.

This work was supported by the Maryland Stem Cell Research Fund, and NIH (NS125350).

Table S1. Primer sequences

| F1 | 5’-CTTTGAGCTCTACTGGCTTCT-3’ |
| --- | --- |
| F2 | 5’-GCTTGGATCCCTCGAGTTAAT-3’ |
| R1 | 5’-GGCTTGTACTCGGTCATCTC-3’ |
| R2 | 5’- CACGTAACCTGAGAAGGGAATC-3’ |
| F3 | 5’-CGGTTAATGTGGCTCTGGTT-3’ |
| R3 | 5’-AGGATCCTCTCTGGCTCCAT-3’ |


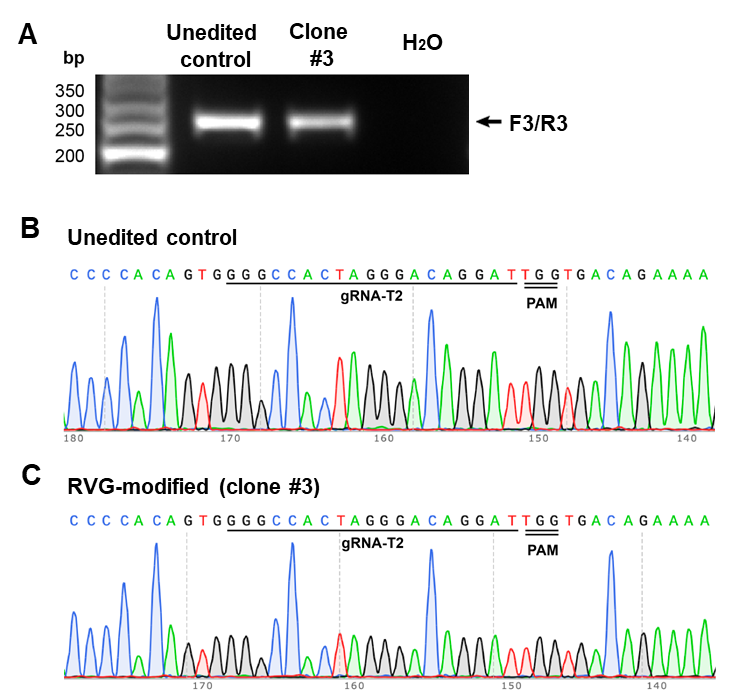


Fig. S1. Characterization of the unedited *AAVS1* allele in the RVG-modified hiPSCs. (A) F3 and R3 primers amplified a PCR band of 254 bp in clone #3, indicating that clone #3 is heterozygous for the integration into the *AAVS1* locus. The location of F3 and R3 primers are shown in Fig. 1A. Unedited control hiPSCs are included as a positive control. (B-C) The PCR bands from A were Sanger sequenced using the F3 primer showing no indels are present in the second allele of *AAVS1* in the RVG-modified clone #3 hiPSCs. gRNA and PAM sequences are underlined.


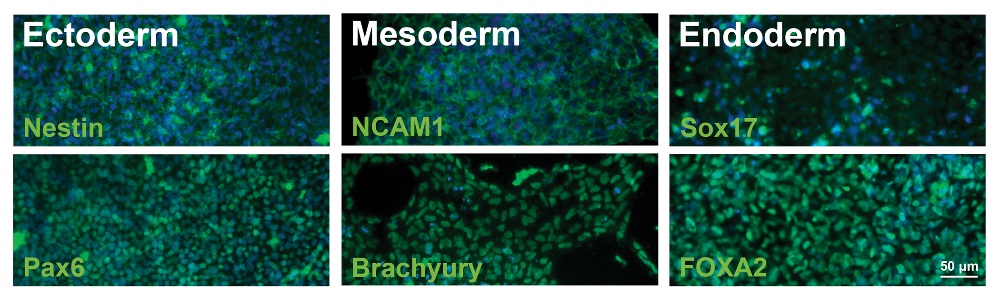


Fig. S2. Trilineage differentiation potential of the RVG-edited hiPSC line. RVG-edited line shows trilineage differentiation potential, as shown by expression of protein markers in ectoderm (Nestin and Pax6), mesoderm (NCAM1 and Brachyury), and endoderm (Sox17 and FOXA2) germ layers, respectively. Nuclei were stained by Hoechst 33342. Scale bar: 50 µm.


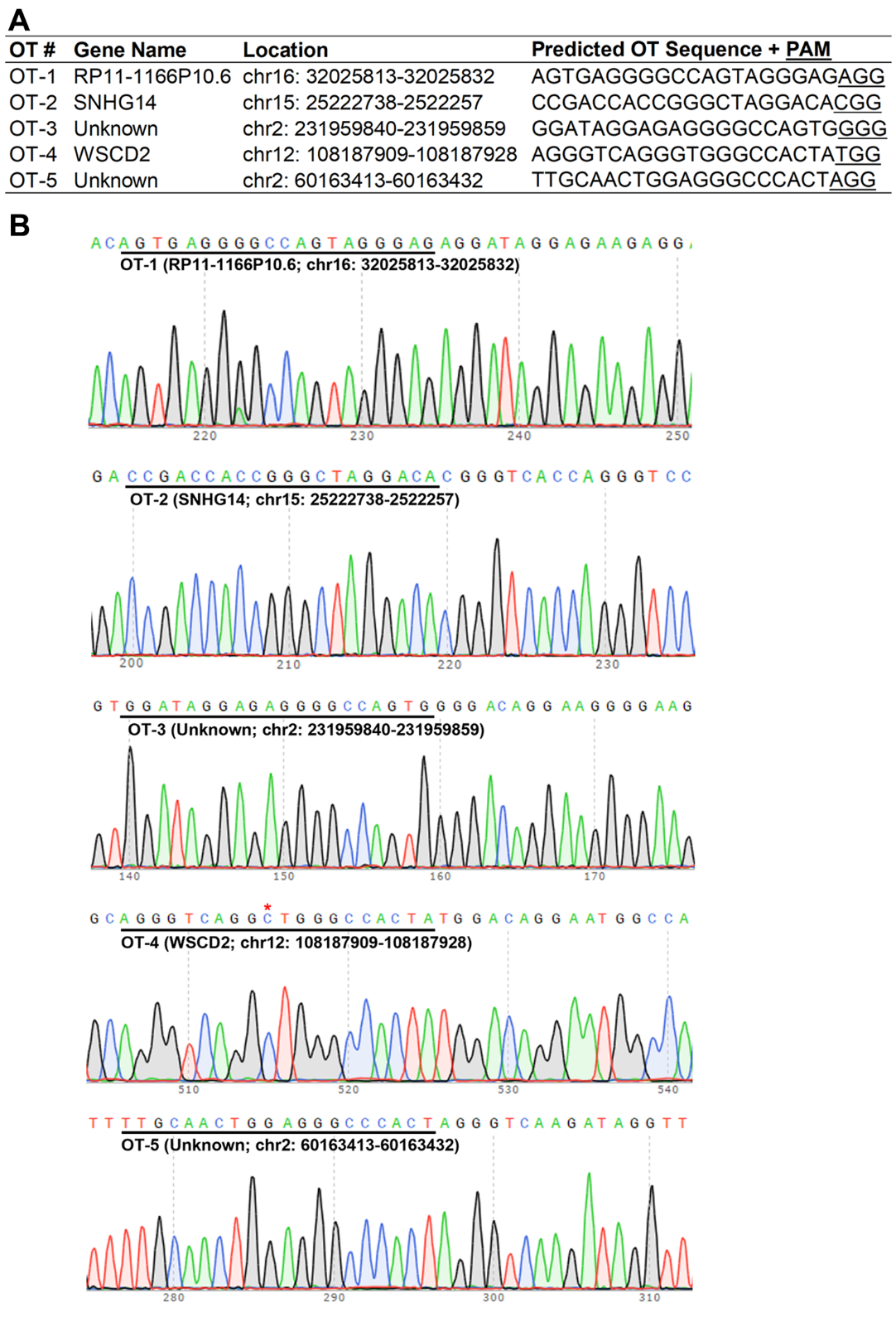


Fig. S3. Genome editing at the *AAVS1* locus does not generate indels in any of the top 5 predicted off-target (OT) sites in the RVG-edited iPSC clone. (A). A table showing the top 5 predicted off-target sites by AAVS1-T2 gRNA. Genomic DNA extracted from the RVG-edited iPSC clone was used for PCR amplification of the 5 sites. (B) Sanger sequencing showed no indels in the PCR products amplified from the top 5 predicted off-target sites.


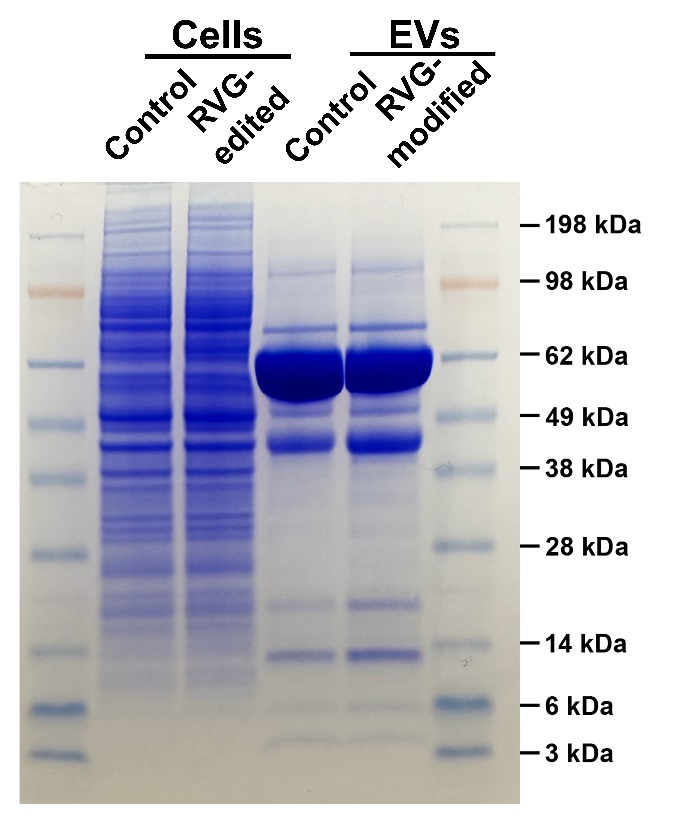


Fig. S4. Coomassie brilliant blue R-250 staining analysis for the iPSCs and EVs in Fig. 2C. EV group differs from the cell group and shows well-defined protein patterns, suggesting little cross contamination between these two sample groups. CD63 is the major marker for exosomes and is the highest EV protein component, indicating high purity of EV samples.


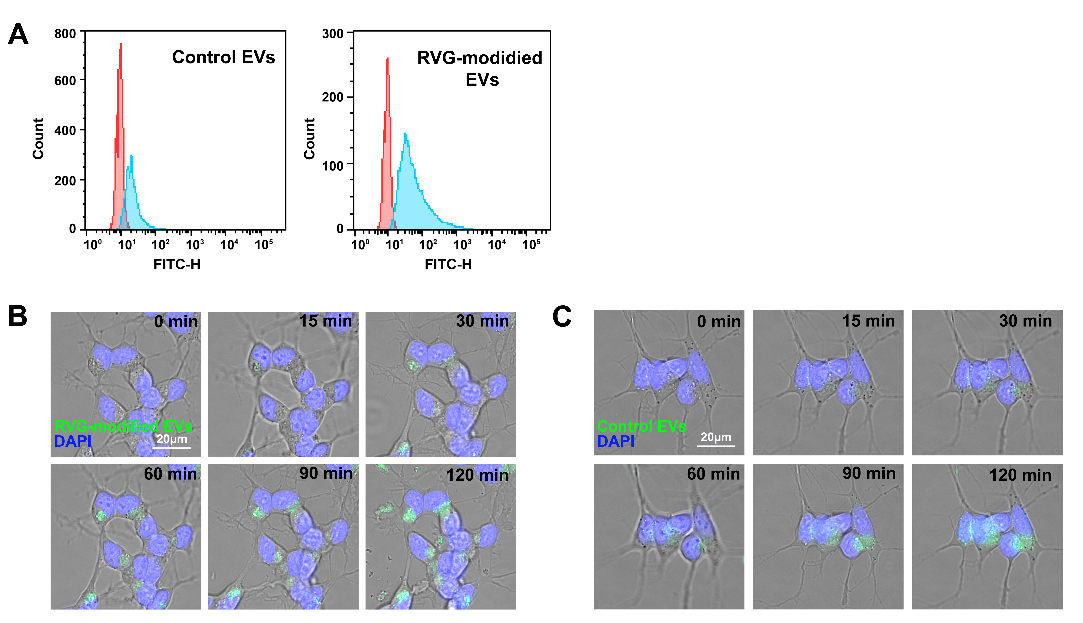


Fig. S5. Labeling and neuronal uptake of control and RVG-modified EVs. (A) EV cargo protein was labeled with the green fluorescence and then EV fluorescence was analyzed using nanoparticle flow cytometry. (B-C) Microscopic imaging of live SH-SY5Y cells up taking the control or RVG-modified EVs. The uptake reaches a plateau at 120 min.


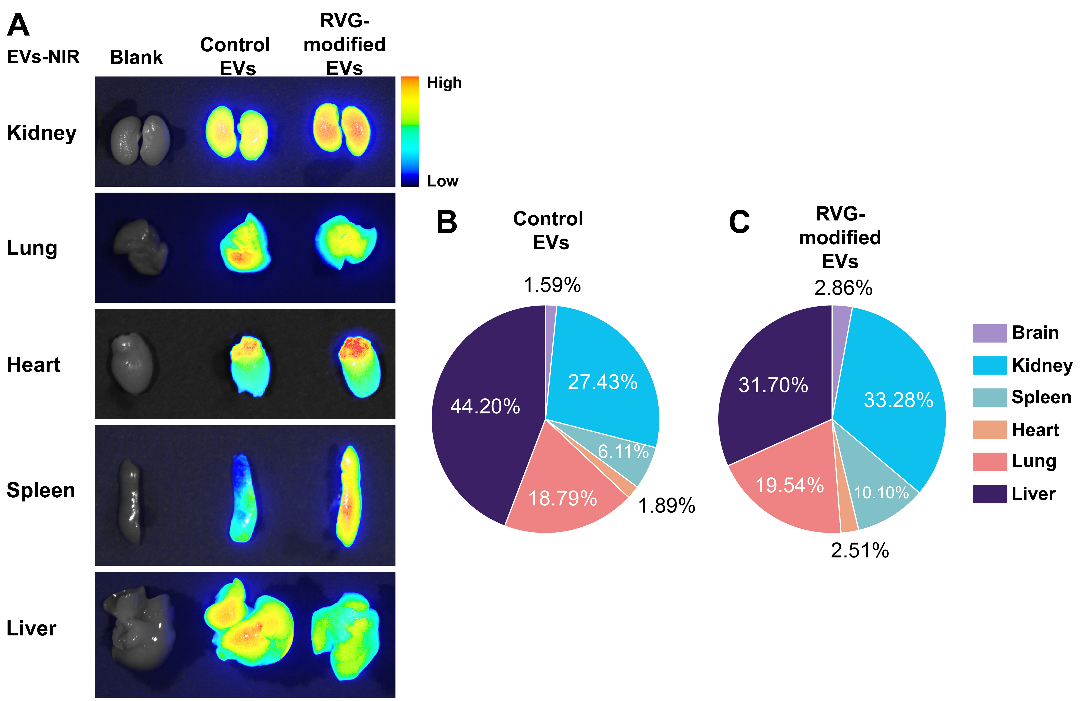


Fig. S6. Biodistribution of RVG-modified EVs. (A) Representative NIR signals of control and RVG-modified EVs in mouse peripheral organs 24 hours post intravenous injection. (B) Quantification of mean fluorescence intensity of RVG-modified EVs in different organs. Data were presented as mean ± SEM (*n* = 3) and analyzed using one-way ANOVA followed by Tukey post hoc test. **p* < 0.05.


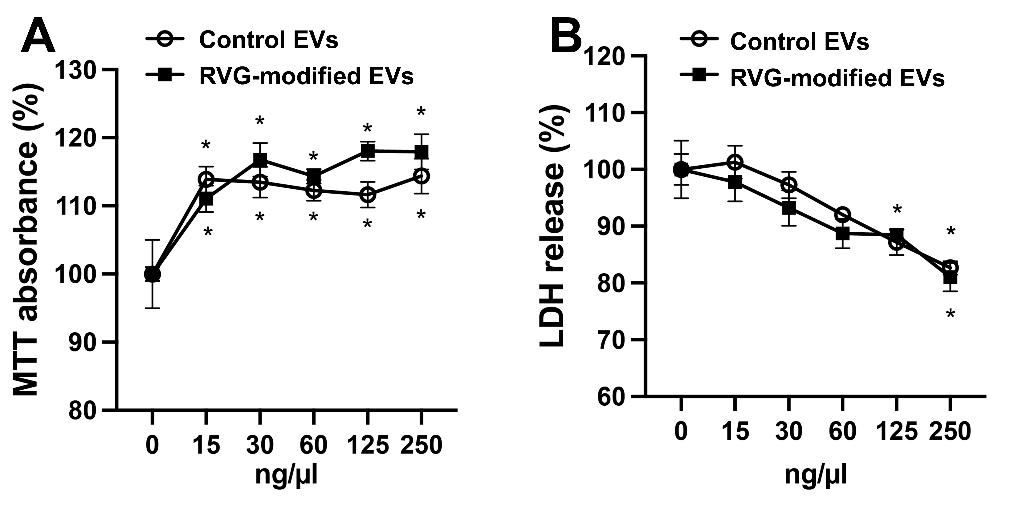


Fig. S7. Labeling and neuronal uptake of control and RVG-modified EVs. (A-B) Cellular metabolism and cell membrane integrity of labeled EVs were measured by [3-(4,5-dimethylthiazol-2-yl)-2,5-diphenyltetrazolium bromide] (MTT, A) and lactate dehydrogenase (LDH, B) release assays, respectively. Data are presented as mean ± SEM (*n* = 6) and analyzed using one-way ANOVA followed by Tukey post hoc test. **p* < 0.05.


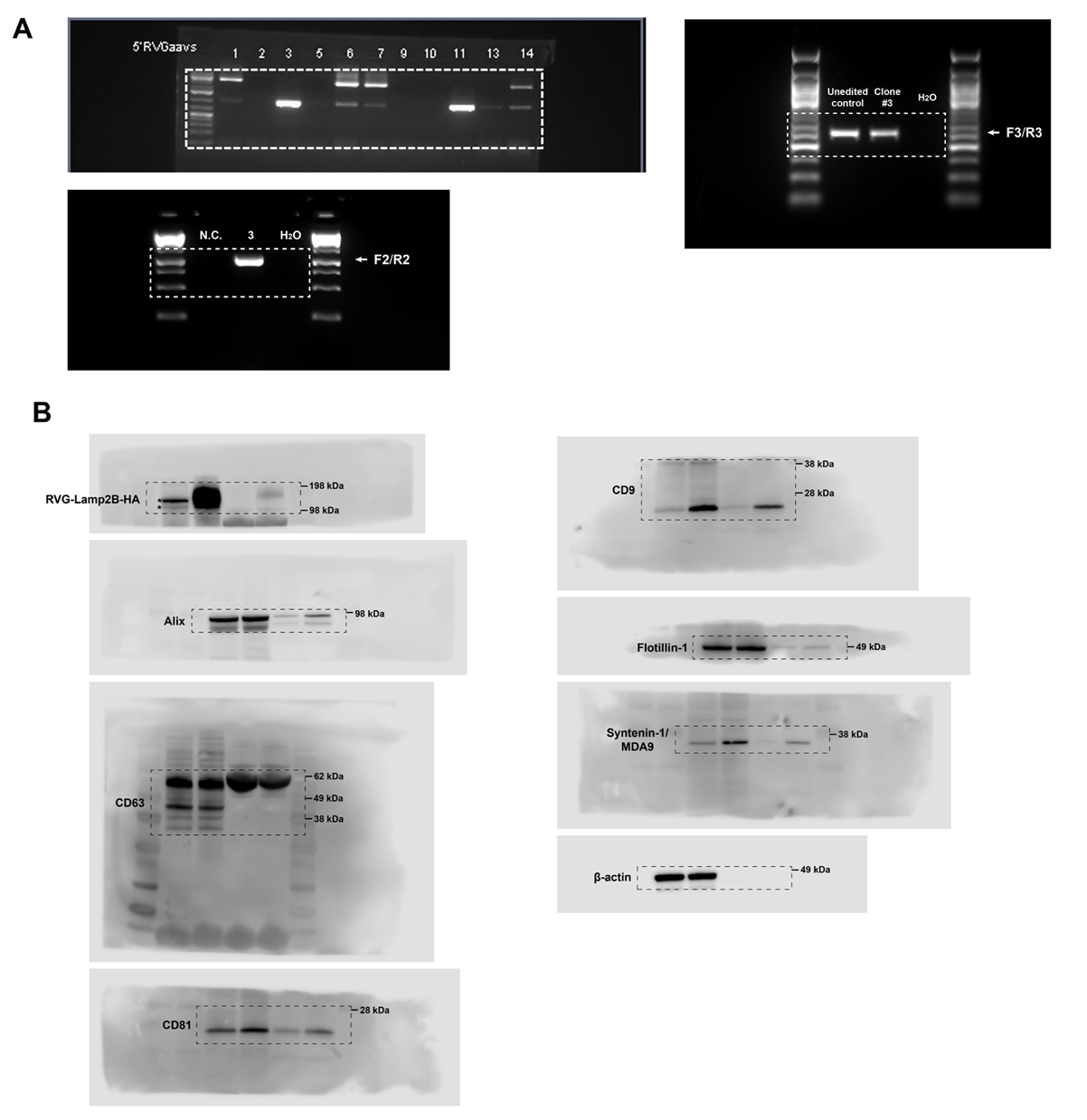


Fig. S8. Uncropped gel images and western blots. The cropping areas are indicated by dashed boxes with ladders.
